# Screening for precancerous anal lesions linked to human papillomaviruses: French recommendations for clinical practice

**DOI:** 10.1007/s10151-023-02899-8

**Published:** 2024-01-10

**Authors:** L. Spindler, I. Etienney, L. Abramowitz, V. de Parades, F. Pigot, L. Siproudhis, J. Adam, V. Balzano, D. Bouchard, N. Bouta, M. Bucau, A. Carlo, J. Chanal, C. Charpentier, G. Clifford, M. Draullette, N. Fathallah, V. Ferré, J.-F. Fléjou, S. Fouéré, T. Higuero, L. Kassouri, S. Kurt, A. Laurain, E. Leclerc, Q. Lepiller, A.-C. Lesage, D. Mège, A. Ménard, P. Merle, P. Mortreux, C. Noël, H. Péré, J.-L. Prétet, D. Roland, G. Staumont, L. Tracanelli, L. Vuitton, S. Wylomanski, O. Zaegel-Faucher, Société Nationale Française de Colo-Proctologie

**Affiliations:** 1 Service de Proctologie, Groupe Hospitalier Paris Saint-Joseph, Paris, France; 2 Service de Proctologie, Hôpital Diaconesses-Croix Saint Simon, Paris, France; 3 Service de Proctologie, APHP Hôpital Bichat-Claude Bernard and Ramsay GDS Clinique Blomet, Paris, France; 4 Service de Proctologie, Hôpital Bagatelle, Talence, France; 5 Service d’Hépato-Gastroentérologie, CHU Pontchaillou, Rennes, France; 6 Service d’Anatomopathologie, Groupe Hospitalier Paris Saint-Joseph, Paris, France; 7 Service de Gastroentérologie et Oncologie Digestive, CHU Tours, Tours, France; 8 Service d’Hépato-Gastroentérologie et de Proctologie, Clinique La Croix du Sud, Quint-Fonsegrives, France; 9 Service d’Anatomopathologie, AP-HP Hôpital Bichat-Claude Bernard, Paris, France; 10 Service de Dermatologie, AP-HP, Hôpital Tarnier, Paris, France; 11 Département de Virologie, AP-HP, Hôpital Bichat-Claude Bernard, INSERM, IAME, Université de Paris, Paris, France; 12 Early Detection, Prevention, and Infections Branch, International Agency for Research On Cancer, Lyon, France; 13 Service d’Hépato-Gastroentérologie et Assistance Nutritive, AP-HP, Hôpital Beaujon, Clichy, France; 14 Service d’Anatomopathologie, Cerbapath, Paris, France; 15 Service de Dermatologie, AP-HP, Hôpital Saint-Louis, Université de Paris, Paris, France; 16 Centre des Maladies Sexuellement Transmises, AP-HP, Hôpital Saint-Louis, Paris, France; 17 Gastro-entérologue, proctologue medico-chirurgical, Beausoleil, France; 18 Service d’Hépato-Gastroentérologie, CHU Clermont-Ferrand, Inserm, 3iHP, Université Clermont Auvergne, Clermont-Ferrand, France; 19 Laboratoire de Virologie, CHU de Besançon, Besançon, France; 20 Service de Chirurgie Digestive, Université d’Aix Marseille, AP-HM, Hôpital de la Timone, Marseille, France; 21 Institut Hospitalo-Universitaire Méditerranée Infection, AP-HM, Hôpital Nord, Université d’Aix Marseille, Marseille, France; 22 Service de Gastroentérologie, Centre Hospitalier de Bethune Beuvry, Beuvry, France; 23 Service d’Hépato-Gastroentérologie, CHU de Brest, Brest, France; 24 Laboratoire de Virologie, Service de Microbiologie, AP-HP, Hôpital Européen Georges Pompidou, Paris, France; 25 Functional Genomics of Solid Tumors (FunGeST), Centre de Recherche des Cordelier, INSERM, Université de Paris, Sorbonne Université, Paris, France; 26 EA3181, Université de Franche-Comté, LabEx LipSTIC ANR-11-LABX-0021, Besançon, France; 27 Centre National de Référence Papillomavirus, CHU de Besançon, Besançon, France; 28 Service de Gastroentérologie, CHU de Besançon, Université de Bourgogne Franche-Comté, Besançon, France; 29 Service de Gynécologie, Groupe Hospitalier Paris Saint-Joseph, Paris, France; 30 Service d’Immuno-Hématologie Clinique, AP-HM, CHU Sainte-Marguerite, Marseille, France

**Keywords:** HPV, Screening, Guidelines, High-resolution anoscopy, Precancerous anal lesions, HSIL

## Abstract

In France, about 2000 new cases of anal cancer are diagnosed annually. Squamous cell carcinoma is the most common histological type, mostly occurring secondary to persistent HPV16 infection. Invasive cancer is preceded by precancerous lesions. In addition to patients with a personal history of precancerous lesions and anal cancer, three groups are at very high risk of anal cancer: (i) men who have sex with men and are living with HIV, (ii) women with a history of high-grade squamous intraepithelial lesions (HSILs) or vulvar HPV cancer, and (iii) women who received a solid organ transplant more than 10 years ago. The purpose of screening is to detect HSILs so that they can be treated, thereby reducing the risk of progression to cancer. All patients with symptoms should undergo a proctological examination including standard anoscopy. For asymptomatic patients at risk, an initial HPV16 test makes it possible to target patients at risk of HSILs likely to progress to cancer. Anal cytology is a sensitive test for HSIL detection. Its sensitivity is greater than 80% and exceeds that of proctological examination with standard anoscopy. It is indicated in the event of a positive HPV16 test. In the presence of cytological abnormalities and/or lesions and a suspicion of dysplasia on clinical examination, high-resolution anoscopy is indicated. Performance is superior to that of proctological examination with standard anoscopy. However, this technique is not widely available, which limits its use. If high-resolution anoscopy is not possible, screening by a standard proctological examination is an alternative. There is a need to develop high-resolution anoscopy and triage tests and to evaluate screening strategies.

## Introduction

In France, just over 2000 new cases of anal cancer are diagnosed annually, and the incidence of this disease is steadily increasing. The male/female sex ratio is 0.3 [1, 2]. Most anal cancers are squamous cell carcinomas (SCCs), associated with human papillomavirus (HPV) infection in 88% to 97% of cases [3], the principal virus implicated being HPV16 [4]. SCCs are preceded by precancerous lesions called squamous intraepithelial lesions. A distinction is made between low-grade squamous intraepithelial lesions (LSILs, formerly p16-negative AIN1 and AIN2) and high-grade squamous intraepithelial lesions (HSILs, formerly p16-positive AIN2 and AIN3) [5].

The groups considered at greatest risk are targeting for the screening of precancerous lesions and SCC (Table 1). Three groups are considered at “very high risk” of anal cancer: (i) men who have sex with men (MSM) and are living with human immunodeficiency virus (HIV) (MSM HIV+), (ii) women with a history of HSIL or vulvar HPV cancer, and (iii) women who underwent solid organ transplantation more than 10 years ago. In these populations, the annual incidence exceeds 40/100,000 (versus 0.8 to 2.5/100,000 for the general population) [6]. Patients with a history of HSIL also warrant close monitoring and are considered to be at very high risk. Screening these “very high-risk” populations is a matter of priority and is the subject of our recommendations.

**Table 1:** Principal risk groups for anal cancer based on [6]

Populations	Annual incidence of anal cancer (per 100,000)
People living with HIV	
Men who have sex with men (MSM)*	85
< 30 years old	17
Between 30 and 45 years old*	> 60
> 45 years old*	≥ 100
Men who have sex with women	32
Women	22
MSM without HIV	19
Solid organ transplant patients (transplantation > 10 years ago)	
Men	24.5
Women*	50
Women with a history of HPV-related precancerous lesions/gynecological cancers	
Precancerous lesions of the cervix (CIN2 and more)	6
Cervical cancer	9
Precancerous lesions of the vagina (VAIN3)	19
Vaginal cancer	10
Precancerous lesions of the vulva (VIN3)*	42
Vulvar cancer*	45
History of autoimmune disease	
Systemic lupus erythematosus	10
Ulcerative colitis	6
Crohn’s disease	3

*Very high-risk groups targeted for screening

The rate of progression of HSILs to SCC varies, even within the populations at risk, and the published data are heterogeneous. In the French AIN3 cohort, the rate of progression was 1.16 per 100 patient-years [95% CI 0.84–1.47] [7]. However, progression is not always unequivocal, and the spontaneous clearance of HSILs can occur. Such clearance can occur in more than 20% of male patients at risk [8]. The factors influencing HSIL progression include the presence of HPV16, patient age, the size of the HSILs, and immunosuppression [7, 8]. The natural course of HSILs remains imperfectly understood, but must be taken into account in the screening, treatment, and monitoring of patients at risk.

## Methods

A panel of 40 French experts (gastroenterologists, colorectal surgeons, infection specialists, virologists, epidemiologists, dermatologists, gynecologists, and pathologists) specializing in HPV infection and its manifestations reviewed the literature with a view to proposing practical management pathways for the screening of precancerous anal lesions due to HPV. Each recommendation is graded, and a management algorithm was designed for screening and monitoring.

All the authors searched the PubMed and Cochrane databases for articles published up to September 2022. An analysis of the literature was performed in accordance with the recommendations of the Haute Autorité de Santé (HAS, the French High Authority for Health), making it possible to grade the recommendations. If an absence of factual data made it impossible to establish a grade of recommendation according to HAS guidelines, then proposals based on expert opinion were drafted. If all the authors agreed on a consensus statement, it was then submitted to all members of the Société Nationale Française de Colo-Proctologie (SNFCP) via e-mail. Scores from 1 to 9 were assigned according to the RAND/UCLA method. The results were analyzed and statements were rewritten, if necessary, by the steering committee for this work. The resulting declarations are referred to as “expert agreements” (EAs).

## Screening for precancerous anal lesions

### Objectives of screening

The phase III randomized controlled study of the North American ANCHOR group [9] showed that the treatment of HSIL reduced the risk of progression to SCC by 57% for people living with HIV (PLHIV) after 25 months of follow-up. There were nine incident cases in the “treatment” group (i.e., 173 per 100,000 person-years, 95% CI 90–332 per 100,000 person-years) and 21 in the “surveillance” group (402 per 100,000 person-years, 95% CI 262–616 per 100,000 person-years) [9]. Moreover, the rates of SCC detection at stage I or II were higher than those reported in national data for the USA [10]. In the French AIN3 cohort, the systematic monitoring of patients with AIN3 led to the detection of 60% of SCCs at stage 1, versus only 15% in real-life conditions [7, 11]. These findings suggest that the monitoring of at-risk groups facilitates the early identification and treatment of SCC.

**Table:** 

The treatment of high-grade precancerous lesions (HSILs) decreases the risk of progression to SCC. *Grade A*
Screening for precancerous anal lesions facilitates the early diagnosis of SCC, suggesting a possibility for increasing survival. *Grade C*

### Screening methods

#### Standard proctological examination

The standard proctological examination (SPE) includes inspection of the anal margin, a digital anorectal examination, and anoscopy. The International Anal Neoplasia Society published recommendations in 2012 for digital anorectal examinations to screen for lesions due to HPV [12]. The SPE is well tolerated, and acceptability rates are high [13–16]. Any patient presenting with proctological symptoms should undergo SPE. This examination can detect warts, macroscopically visible HSILs, and SCC at an early stage. Conversely, digital anorectal examination has a poor performance for the detection of HSILs [14]. In a prospective series of 446 men living with HIV, digital anorectal examination failed to detect any of the 156 cases of HSIL [17]. Studies on the performance of the SPE are scarce. The strategies most frequently compared are anorectal examination, cytology, and high-resolution anoscopy (HRA). In a study reporting the results of proctological examinations on 1206 PLHIV, 26% were found to have an HPV-related anal lesion, which was limited to the anal canal in 52.7%: 9.2% had a lesion with no dysplasia, 10.2% had an LSIL, 6% an HSIL, and 0.6% cancer [14]. Another study compared different screening strategies in 212 PLHIV. The diagnostic performance of a combination of SPE, anal smear, and HR-HPV test for the detection of HSIL was significantly better than that of SPE alone, with detection rates of 12.7% versus 3.3% [18]. Finally, several studies have shown that HRA is more effective than clinical examination for HSIL screening [18–22]. Camus et al. showed that 65.7% of the HSILs detected by HRA were not visible to the naked eye [22].

**Table:** 

1. Conditions for performing the SPE
Symptoms, such as anal pain, swelling, and bleeding, should be checked for during the interview and, if present, a proctological examination should be performed by a gastroenterologist/proctologist. *Grade C*
Examinations of the anal margin and digital anorectal examinations can be performed by any doctor. *Grade C*
The training of patients/their partners and doctors who are not proctologists in screening makes it possible to increase the dissemination, implementation, and performance of screening. *Grade B*
Any gastroenterologist in France should be able to perform a complete proctological examination with standard anoscopy. Training in how to perform this examination and in screening for anal cancer should be an integral component of both initial and on-the-job training. *Expert agreement*
Anorectal and complete proctological examinations are rapid, cheap, and well accepted by patients. *Grade C*
Warts are mostly lesions without dysplasia or with low-grade dysplasia because they are linked to low-risk HPV. HSILs visible to the naked eye have a variable macroscopic appearance in terms of size, color, and shape. Any suspicious lesions should be biopsied to check for invasive SCC. *Grade C*
The benefit of acetic acid during a standard proctological examination has not been demonstrated. *Grade C*
Collaboration between the doctors following the populations at risk of anal cancer and gastroenterologists/proctologists must be strengthened. Each care center should define a referent proctologist if possible. *Expert agreement*
2. Diagnostic performance
Detecting anal cancers at an early stage is an important goal of screening. *Grade B*
Examination of the anal margin and digital anorectal examination can detect cancers of the anus at an early stage. *Grade B*
A complete proctological examination with standard anoscopy can detect warts, macroscopically visible HSILs, and anal cancers at an early stage. *Grade C*
Screening strategies should take the availability of different screening tools into account. *Grade C*
3. Strategic limitations
The benefit of standard anoscopy-based screening for decreasing the incidence of anal cancer has not yet been demonstrated. *Grade B*
HSILs cannot be diagnosed by digital anorectal examination. *Grade A*
Clinical examination with standard anoscopy has low sensitivity for the diagnosis of HSIL. Less than 40% of HSILs are visible to the naked eye. *Grade A*

#### Anal cytology

An anal smear test involves collecting transitional cells by introducing a swab into the anal canal without prior disinfection or lubrication, at least 48 h after last sexual intercourse, in the absence of any local treatment or infection [23, 24]. Anal self-sampling (ASS) is a possible alternative, given its acceptability, feasibility, and similar diagnostic performance [25, 26]. Anal cytology should be performed only if referral for HRA is possible.

The diagnostic performance of cytology is better for the detection of histological HSILs (hHSIL) than the SPE [18]. However, this performance varies according to the population studied. In their meta-analysis, Clarke et al. found that anal cytology had a sensitivity of 81% at and above (ASC-US+) the ASC-US threshold and a specificity of 62.4% [27]. This performance is close to that of cervical smear tests [28]. For HIV+ MSM, a sensitivity of 85.2% has been reported, with a specificity of 52.8%, and a false-negative rate of 6%. The SPANC study showed that the specificity of cytology increases with age and that sensitivity increases with the size and number of HSILs [8]. In women, a sensitivity of 65.7% has been reported, with a specificity of 82.2%, and a false-negative rate of 5% [27]. Thus, for HIV+ women and MSM, anal cytology has an excellent negative predictive value. Finally, among MSM not living with HIV (HIV− MSM), cytology has been shown to have a sensitivity of only 56.6% and a specificity of 66.5%. The sensitivity of anal cytology therefore appears to be higher in HIV+ MSM. The main limitation of cytology is its lack of specificity, which can lead to HRA being performed unnecessarily. Regardless of the population, there is only a weak correlation between the grade of cytological abnormalities and the grade of histological abnormalities [29]. In cytological examinations detecting ASC-US and LSILs, the frequency of hHSIL is non-negligible [30–32]. In the meta-analysis performed by Clarke et al., the risk of hHSIL in cases of ASC-US+ abnormalities was 35%. Thus, the detection of low-grade cytological abnormalities does not rule out the presence of hHSILs. Conversely, the presence of high-grade cytological abnormalities appears to be strongly predictive of hHSIL, with a sensitivity of 21.1% and a specificity of 96.4%. Diagnostic performance, therefore, varies with the type of lesion detected (ASC-US, LSIL, or HSIL).

The combination of HPV testing and cytology appears to improve sensitivity at the expense of specificity, due to the high prevalence of high-risk HPV infection (HR-HPV) in at-risk groups [31, 33–35]. The combination of SPE, anal cytology, and tests for HPV16 increases the rate of hHSIL detection [18]. Dual-staining for p16 and Ki67 may improve the performance of anal cytology, but insufficient data are currently available to confirm this [27, 36].

**Table:** 

1. Conditions for performing anal cytology screening
Anal cytology screening should be performed in target populations. *Grade A*
The evidence for benefits of proctological screening is better for PLHIV aged ≥ 35 years. *Grade A*
There are insufficient data to determine the exact rate at which surveillance by anal cytology should be performed. *Grade C*
2. Diagnostic performance
Anal cytology is twice as sensitive as clinical examination for the detection of HSILs. *Grade B*. However, its specificity is limited. *Grade A*
The grade of cytological abnormalities is poorly correlated with the grade of histological abnormalities. *Grade B*
The anal cytology screening strategy is indicated for high-risk populations at expert centers, but the sensitivity and specificity of the test are insufficient to justify its use for mass screening. *Grade B*
A complete strategy, defined as a combination of standard anoscopy, anal cytology, and HPV16 detection, provides higher detection rates for HSILs. *Grade B*
Most international recommendations are based on the performance of an anal smear followed by HRA if an abnormality is found. Such strategies have been the most studied, but possible association with an HPV test could lead to their evolution. *Grade C*
3. Strategic limitations
Anal cytology is of marked interest in the absence of visible lesions on clinical examination. In cases of macroscopic lesions, targeted biopsies are indicated for histological analysis. *Grade B*
Anal cytology has a high sensitivity but a low specificity, leading to the risk of performing “unnecessary” HRA. *Grade A*
HRA must be performed at an expert center in the event of abnormal cytological results for patients from populations at a high risk of cancer. *Grade B*
If HRA is unavailable, the goal of screening should be the early diagnosis of anal cancer and macroscopic HSILs. *Expert agreement*

#### HR-HPV screening

HR-HPV screening is based on detection of the viral genome by PCR. The intra-anal swab sample should be taken from the transitional zone [37, 38]. Several studies have demonstrated the feasibility and acceptability of ASS among HIV+ MSM [25]. Another study assessed the performance of ASS in 300 women in Zimbabwe, half of whom were living with HIV. Moderate concordance was found between the results of smears for detecting and typing HPV obtained by ASS and those for the sample taken by a clinician (kappa = 0.55). The proportions of the HR-HPV types detected did not differ significantly according to the sampling method [39]. ASS may, therefore, be a suitable alternative strategy for facilitating the expansion of screening within the populations for which screening is recommended, but further studies are needed.

The test for HR-HPV is extremely sensitive for the detection of HSILs, with a sensitivity of 90% [27]. Conversely, its specificity is low, at 47% for women and only 35% for HIV+ MSM, due to the particularly high prevalence of HR-HPV infection, particularly in HIV+ MSM. One study reported a sensitivity of 75% and a specificity of 86% for the detection of HSILs in HIV+ women. HPV16 detection was also found to be very strongly predictive of HSIL lesions [40]. Another study reported similar sensitivities for HSIL detection by cytology and by the HR-HPV test, with a 67% higher specificity for the HR-HPV test than for cytology [41]. In cases in which the HR-HPV test is negative, the probability of HSIL has been estimated at 4% [27].

Restricting testing to HPV16 (rather than all HR-HPVs) decreases sensitivity, but clearly increases specificity [27, 42]. In their meta-analysis, Clarke et al. found a sensitivity of HPV16 testing for the general population of 46% and a specificity of 83%. Among HIV+ MSM, the corresponding values were 42.4% and 80.4%, respectively. However, the false-negative rate was high, at 16%. A negative HPV16 test does not, therefore, rule out the presence of HSILs [27]. However, HPV16 infection is strongly associated with SCC and is a major risk factor for the progression of HSIL to SCC [4, 7, 8, 43]. Tests for HPV16 could, therefore, make risk stratification possible, even for patients at risk. Finally, the natural course of HPV16 infection and its possible clearance justify the performance of surveillance test at 5-year intervals in the absence of signs of HPV16 infection. Indeed, Alberts et al. reported a cumulative incidence of HPV16 infection of 16% at 3 years in at-risk populations initially free of HPV16 [44]. In France, the HR-HPV test on anal swabs is not reimbursed.

**Table:** 

1. Conditions for HR-HPV testing
The entire anal canal should be swabbed, especially the anorectal transformation zone. *Grade B*
The storage of samples collected for virological purposes in a liquid cytology medium makes it possible to perform anal cytology on the same sample. *Grade A*
The diagnostic performance of self-sampling has yet to be assessed. *Grade C*
2. Diagnostic performance
For patients at risk, HR-HPV testing is sensitive, but not very specific for the diagnosis of HSIL, due to a high prevalence of anal HR-HPV infection. *Grade A*
In the event of a positive HR-HPV test, a specific triage test should be considered. *Grade C*
The combination of HPV testing and anal cytology improves diagnostic performance. *Grade A*
The restriction of testing to HPV16 improves specificity but decreases sensitivity for the diagnosis of HSIL. *Grade A*
In the absence of anal HR-HPV infection, the probability of developing HSIL lesions within 5 years is low. *Grade B*
3. Strategic limitations
In addition to detecting HR-HPV infection, the persistence of this infection must be evaluated. *Grade C*
The presence of HPV16 is a risk factor for the progression of HSILs to cancer. However, limiting testing to HPV16 markedly decreases the sensitivity of the test for HSIL diagnosis. *Grade B*
In France, tests for HR-HPV in anal swabs are not reimbursed

#### High-resolution anoscopy

Quality criteria have been defined for HRA [45]. It is recommended that practitioners perform at least 50 HRA per year and achieve detection rate of 90% for HSIL in patients with HSIL detected on a smear in the preceding 3 months. The rate of HSIL detection increases with the number of HRAs performed [46]. The learning curve is long [47]. The examination is well tolerated, with an average VAS of 2/10 when performed for diagnostic purposes and 3/10 when the procedure includes a therapeutic maneuver, but pain remains problematic (VAS > 7/10) for 6% of men and 14% of women [48].

Published values for the sensitivity of HRA range from 59% to 100%, whereas specificity ranges from 66% to74% [49]. Camus et al. showed that 65.7% of the high-grade lesions detected by HRA were not visible to the naked eye [22]. The presence of high-grade cytological abnormalities is predictive of a high-grade histological lesion [50]. HRA revealed the presence of AIN2+ lesions in 64% of patients with an HSIL detected on a smear [27]. However, normal or low-grade anal cytology cannot rule out the presence of hHSIL. A longitudinal study of 368 asymptomatic MSM who had undergone successive HRA examinations over a mean of 4 years showed that 11% of HSILs identified by HRA on biopsies coincided with normal smear test results [21]. In France, the APACHES study showed that 3.4% of 524 HIV MSM with HSIL diagnosed by HRA had a normal smear test and that 11.3% had a smear test result classified as ASC-US+ or LSIL [51]. These data confirm the poor concordance between the grade of cytological abnormalities and histological grade.

HRA can be difficult to perform in some patients and its use is limited in certain situations: scarred anus, stenosis, inflammation, history of radiotherapy, etc. In addition, the diagnostic performance of HRA is affected by significant interoperator variability. The APACHES study reported HSIL detection rates ranging from 5.1% to 31.3%, depending on the center, highlighting the need for rigorous training in the practice of HRA [51]. Access to HRA is limited in France (fewer than 30 doctors perform this examination). It is not currently possible to screen all patients at risk, from the outset, by HRA. HRA is a second-line examination mostly performed after an abnormal cytology result is obtained. The development of observer-independent visualization tools based on artificial intelligence algorithms would enhance diagnostic performance and promote learning [52]. Finally, HRA makes the targeted treatment of HSILs possible [9]. However, the risk of HSIL recurrence after treatment is high (49–53% at 1 year and 66–77% at 3 years for HIV− and HIV+ MSM, respectively) and repeat examinations are therefore required [53].

**Table:** 

1. Conditions for performing HRA
HRA is a second-line screening tool. It is performed after an abnormal triage test result, usually following an abnormal anal cytology result. *Grade B*
HRA is also used for the targeted treatment and monitoring of HSILs. *Grade B*
A diagnosis of HSIL is suspected in patients with a combination of staining abnormalities, abnormal vessels, and epithelial changes. Histological confirmation should systematically be sought. *Grade B*
The quality criteria for this examination are the subject of recommendations from the International Anal Neoplasia Society (IANS). *Expert agreement*
Practitioners should perform at least 50 HRAs per year. *Grade C*
2. Diagnostic performance
HRA is the gold standard for the detection, targeted treatment, and monitoring of HSILs. *Grade B*
HRA outperforms clinical examination with standard anoscopy for the diagnosis of HSIL. *Grade B*
It is recommended to ensure that a detection rate for high-grade lesions of 90% is obtained for patients with an HSIL-positive smear in the preceding 3 months. *Grade C*
Due to the poor correlation between anal cytology and histology results, a non-negligible proportion of patients (> 10%) with an ASC-US+ or LSIL smear have high-grade HRA lesions. *Grade B*
3. Strategic limitations
The natural course of high-grade lesions visible only by HRA is unknown. *Expert agreement*
The long learning curve and poor availability of this examination currently limit access to this technique. *Expert agreement*
HRA should be performed by clinicians who have received appropriate training. *Grade B*
When available, HRA should be offered to “at-risk” patients. The efficacy of strategies including HRA has been evaluated principally in MSM patients living with HIV. *Grade B*
The benefit of HRA for decreasing the incidence of anal cancer has yet to be demonstrated. *Grade B*

#### Other biomarkers

The staining of anal biopsy specimens for p16^INK4a^ (p16) and Ki-67 can help to stratify anal precancerous lesions by differentiating HSIL from LSIL [54–58]. However, the prognostic value of these markers has yet to be established [59]. The immunocytochemical technique of p16/Ki-67 dual-staining could improve the performance of anal cytology, but the data currently available are insufficient to determine its utility [36, 60].

In the meta-analysis by Clarke et al., six studies assessed E6/E7 messenger RNA (mRNA) as markers for detecting precancerous anal lesions by two different techniques [27]. The sensitivity was only 74.2%, with a high false-negative rate of 16% for HSIL diagnosis. The specificity was 64.3% [27]. The small number of studies and their heterogeneity highlight the need to continue the evaluation of these markers.

Finally, methylation (host genome and viral DNA) markers are promising in terms for triage purposes, to identify high-grade anal lesions with various degrees of risk of progression to cancer [61–63]. The study of two methylation markers (ZNF582 and ASCL1) in a cross-sectional analysis of smears from the French AIN3 cohort revealed an association between the hypermethylation of these markers on a smear at a given time and a high risk of progression to SCC [7]. The evaluation of these markers should be continued.

**Table:** 

The objective of biomarker use is to improve the specificity of anal cytology and the detection of HR-HPV. *Grade B*
P16/Ki-67 dual-staining
1. Conditions for performing p16/Ki-67 dual-staining
Dual-staining for p16/Ki-67 can be performed on cytological or histological samples
2. Diagnostic performance
The p16 marker can be used to differentiate LSIL from HSIL histologically. *Grade A*
The diagnostic performance of dual-staining for p16 and Ki-67 in immunocytochemistry is not superior to that of cytology alone for the diagnosis of HSIL. *Grade C*
p16/Ki-67 dual-staining is less sensitive but more specific than testing for HR-HPV for the diagnosis of HSIL. *Grade C*
3. Strategic limitations
The diagnostic value of dual p16/Ki-67 dual-staining alone or in combination with anal cytology has not been demonstrated. *Grade C*
Staining for p16 may be of prognostic value but this requires confirmation. *Grade C*
Methylation
1. Conditions for methylation marker use
Methylation markers can be analyzed on cytological or histological samples
2. Diagnostic performance
It is not possible to assess the diagnostic performance of methylation markers on the basis of the data currently published. *Grade C*
Methylation markers may be of prognostic value for predicting the progression of HSIL to cancer. *Expert agreement*
3. Strategic limitations
The diagnostic and prognostic values of methylation markers have yet to be demonstrated. Studies are underway. *Grade C*
E6/E7 mRNA
1. Conditions for E6/E7 mRNA use
The presence of mRNA encoding the E6/E7 oncoproteins is a sign of active HR-HPV infection
These transcripts are sought in cytological samples
2. Diagnostic performance
The diagnostic performance of testing for the presence of E6/E7 mRNA is not superior to cytology alone for the diagnosis of HSIL. *Grade C*
3. Strategic limitations
The diagnostic value of testing for the presence of E6/E7 mRNA alone or in combination with anal cytology has not been demonstrated. *Grade C*

### Algorithm


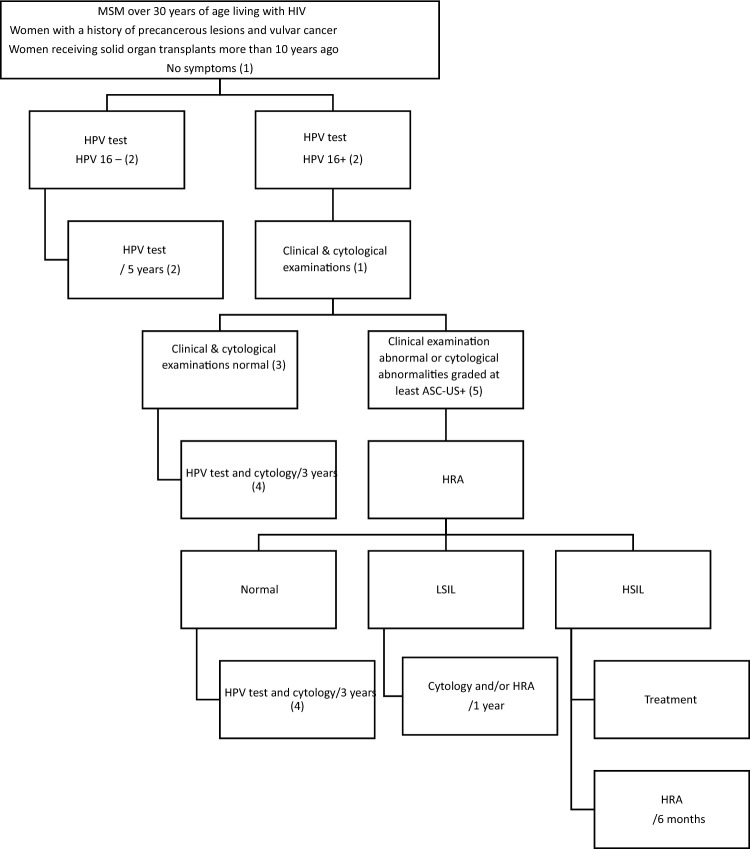

1. SPE is essential if the patient presents proctological symptoms.
2. The search for viral signs of HPV16 infection is at the heart of triage tests because this virus is associated with SCC of the anal canal with a very high prevalence. This strategy limits the need for specialist consultation. The natural course of HPV16 infection and its potential clearance make it possible to perform surveillance tests at 5-year intervals in the absence of signs of HPV16 infection. The cumulative incidence of HPV16 infection is 16% at 3 years for populations at risk without prior HPV16 infection [44].
3. On the basis of current knowledge, a normal cytological examination result renders the presence of HSIL very unlikely (4%).
4. In the presence of HPV16 infection and a normal clinical examination, clearance of the virus is slow and observed in only just over one-third of patients after 3 years of follow-up [44, 64]. It does not, therefore, appear useful to repeat testing at too short an interval in this population, especially if the results of the clinical examination are normal. A normal cytological examination result renders the occurrence of HSIL within 3 years unlikely.
5. The presence of cytological abnormalities is associated with HSIL in one-third of all cases, and two-thirds of cases of high-grade cytological lesions. Pending more discriminating non-invasive tests (methylation test, P16/Ki-67 staining), HRA should be performed under optimal conditions, with biopsies. If HRA is not possible, a SPE must be repeated at least once annually.

**Table:** 

1. Usable strategies and diagnostic performance
The most widely used triage tests are virological tests to detect HR-HPV and liquid-phase anal cytology. The first of these methods is the most sensitive (4% false negatives) and the second is the most specific (64% true positives in the presence of HSIL). The use of these two tests is recommended in patients with risk factors for dysplasia and cancer. *Grade A*
In theory, initial use of an HPV test (with HPV16 genotyping) could be preferred, with positive results leading to the performance of anal cytology. *Grade B*
Other specific tests have not been adequately studied and cannot yet be recommended in routine practice. *Grade B*
2. Strategic limitations
Given the intercenter and interobserver variability of the diagnosis of anal dysplasia, it is recommended that the practitioners and pathologists responsible for making positive diagnoses of dysplasia undergo prior training and compare their diagnostic performances. *Grade B*
The testing of screening protocols in real-life conditions is recommended, to analyze the compliance of at-risk individuals and the conditions for reimbursement of the tests. *Grade C*
Within the framework of screening for the early diagnosis and monitoring of lesions of dysplasia, the management and reimbursement of virological tests and cytological analyses by public authorities is important, as is the specific consideration of HRA

## Conclusion

The incidence of SCC of the anus is steadily increasing. Screening for precancerous anal lesions is a public health issue. The objective should no longer be solely the early diagnosis of cancer but rather the prevention of cancer through the diagnosis and treatment of precancerous anal lesions. Screening is targeted to groups at risk. The performance of a first-line test for HPV16 makes it possible to select the patients for whom the risk of precancerous lesions progressing to SCC is highest, even with the population of high-risk patients. HRA occupies a central position in the detection and management of patients. Its development must be a matter of priority. Further evaluations of triage tests and biomarkers, such as p16/Ki67 and E6/E7 mRNA, and the development of prognostic markers, such as methylation markers, are required. Along with the screening of asymptomatic patients, it is essential for all patients presenting proctological symptoms to undergo a complete proctological examination. In any case, screening strategies warrant further evaluation.

## Data Availability

All data generated or analysed during this study are included in this published article [and its supplementary information files].

## Abbreviations

ASS: Anal self-sampling
EA: Expert agreement
hHSIL: Histological HSIL
HIV: Human immunodeficiency virus
HPV: Human papillomavirus
HRA: High-resolution anoscopy
HR-HPV: High-risk HPV
HSIL: High-grade intraepithelial lesion
LSIL: Low-grade intraepithelial lesion
MSM: Men who have sex with men
PLHIV: Person living with HIV
SCC: Squamous cell carcinoma
SNFCP: Société Nationale Française de Colo-Proctologie
SPE: Standard proctological examination
